# Poster Session II - A275 ENDOTHELIAL-INTRINSIC NOD2 SIGNALLING CONTROLS INTESTINAL EFFECTOR AND MEMORY T CELL GENERATION

**DOI:** 10.1093/jcag/gwaf042.275

**Published:** 2026-02-13

**Authors:** B K Tsankov, M Denney, J S Ahn, C Carr, N Nathan, D Tsang, G Bayer, D J Philpott

**Affiliations:** Immunology, University of Toronto, Toronto, ON, Canada; Immunology, University of Toronto, Toronto, ON, Canada; Immunology, University of Toronto, Toronto, ON, Canada; Weill Cornell Medicine, New York, NY; Immunology, University of Toronto, Toronto, ON, Canada; Immunology, University of Toronto, Toronto, ON, Canada; Immunology, University of Toronto, Toronto, ON, Canada; Immunology, University of Toronto, Toronto, ON, Canada

## Abstract

**Background:**

The generation of intestinal effector and memory T cell responses is critical for protection against enteric infection, and the maintenance of intestinal homeostasis. Indeed, aberrant memory T cell dynamics have been observed in both forms of inflammatory bowel disease, Crohn’s disease (CD) and ulcerative colitis, thereby linking dysregulated control of enteric infection to disease pathology. Moreover, we have previously shown that NOD2 – deficiencies in which form the largest genetic risk factor for CD development – is critical for establishing optimal systemic memory T cell responses. In the present study, we asked what role NOD2 plays in the generation of memory T cell responses in the small intestine.

**Aims:**

1. Address whether NOD2 affects T cell priming in intestinal-draining mesenteric lymph nodes

2. Assess if NOD2 affects effector and memory T cell generation and function in the small intestine

3. Understand the mechanisms by which NOD2 affects T cell priming in the mesenteric lymph nodes and effector and memory functions in the small intestine

**Methods:**

We used a combination of single-cell RNA sequencing, confocal microscopy, adoptive transfer experiments, and acute LCMV infections in WT and *Nod2*^*-/-*^ mice to track the localization, trafficking, and function of LCMV-specific T cells from priming in the intestinal-draining mesenteric lymph nodes (mLN) to effector and memory functions in the small intestinal lamina propria.

**Results:**

We found that NOD2 engagement significantly increases LCMV-specific CD4^+^ T cells in the mLN following infection. ScRNAseq of stromal cells within the mLN revealed high NOD2 expression within blood endothelial cells, with engagement leading to a signature of lymphocyte recruitment. Validation experiments revealed a role for endothelial-cell NOD2 in T cell homing to the mLN, through induction of chemokines and adhesion molecules. Analyses of LCMV-specific T cells in the small intestinal lamina propria at the effector and memory stages of LCMV infection revealed a dramatic reduction of T cells, and a decreased ability to clear secondary infection in this compartment in NOD2-deficient contexts.

**Conclusions:**

We found an unexpected role for endothelial-intrinsic NOD2 in mediating optimal effector and memory T cell responses in the small intestine. Our results indicate that CD patients with *NOD2* polymorphisms may have a decreased ability to clear enteric infections.

**Funding Agencies:**

CAG, CIHR

